# Identification of Metastasis-Associated MicroRNAs in Metastatic Melanoma by miRNA Expression Profile and Experimental Validation

**DOI:** 10.3389/fgene.2021.663110

**Published:** 2021-04-09

**Authors:** Yunshu Gao, Jiahua Xu, Hongwei Li, Yi Hu, Guanzhen Yu

**Affiliations:** ^1^Department of Oncology, People's Liberation Army (PLA) General Hospital, Beijing, China; ^2^Department of Oncology, Longhua Hospital Affiliated to Shanghai University of Traditional Chinese Medicine, Shanghai, China

**Keywords:** miRNA, miR-18a-5p, miR-155-5p, MiR-93-5p, melanoma, metastasis

## Abstract

It is reported that microRNAs (miRNA) have paramount functions in many cellular biological processes, development, metabolism, differentiation, survival, proliferation, and apoptosis included, some of which are involved in metastasis of tumors, such as melanoma. Here, three metastasis-associated miRNAs, miR-18a-5p (upregulated), miR-155-5p (downregulated), and miR-93-5p (upregulated), were identified from a total of 63 different expression miRNAs (DEMs) in metastatic melanoma compared with primary melanoma. We predicted 262 target genes of miR-18a-5p, 904 miR-155-5p target genes, and 1220 miR-93-5p target genes. They participated in pathways concerning melanoma, such as TNF signaling pathway, pathways in cancer, FoxO signaling pathway, cell cycle, Hippo signaling pathway, and TGF-beta signaling pathway. We identified the top 10 hub nodes whose degrees were higher for each survival-associated miRNA as hub genes through constructing the PPI network. Using the selected miRNA and the hub genes, we constructed the miRNA-hub gene network, and PTEN and CCND1 were found to be regulated by all three miRNAs. Of note, miR-155-5p was obviously downregulated in metastatic melanoma tissues, and miR-18a-5p and miR-93-5p were obviously regulated positively in metastatic melanoma tissues. In validating experiments, miR-155-5p's overexpression inhibited miR-18a-5p's and miR-93-5p's expression, which could all significantly reduce SK-MEL-28 cells' invasive ability. Finally, miR-93-5p and its potential target gene UBC were selected for further validation. We found that miR-93-5p's inhibition could reduce SK-MEL-28 cell's invasive ability through upregulated the expression of UBC, and the anti-invasive effect was reserved by downregulation of UBC. The results show that the selected three metastasis-associated miRNAs participate in the process of melanoma metastasis via regulating their target genes, providing a potential molecular mechanism for this disease.

## Introduction

As the neoplasm of the cells, melanoma starts in skin cells called melanocytes (McComiskey et al., 2015). Environmental factors, for example, ultraviolet light exposure, are considered as the main cause of melanoma (Kanavy and Gerstenblith, 2011). This tumor is predominant in the skin or adjacent to the skin and spread throughout the body (Bakkal et al., 2015), with a dramatically increased global incidence over the past few decades (Azoury and Lange, 2014). Moreover, there are still no satisfactory treatments for patients with advanced melanoma because of its complex pathogenesis. Like other types of malignant tumors, the main cause of melanoma-related deaths is metastasis (Shaikh et al., 2016). Thus, the pathogenesis and inhibition of metastasis have become a focal point of melanoma.

Recently, microarray technology has been widely used in investigating gene alterations in metastasis, tumorigenesis, drug resistance, and cancer recurrence, as well as to identify biomarkers for tumor diagnosis, prognosis, and therapy (Hu et al., 2010; Chien et al., 2015; Tabaries et al., 2015). With the next-generation sequencing technology's experimental application, Gene Expression Omnibus (GEO) collects data exponentially and gradually plays an important role in bioinformatics analysis (Barrett et al., 2013). Increasing reliable and functional miRNAs, as reported, have a vital influence on melanoma initiation, progression, and recurrence (Lou et al., 2018). DNA chip-based sequencing technology analyzes TP53 germline mutations in pediatric tumor patients, and simultaneously analyzes all coding exons of TP53 (Harris and McCormick, 2010). The entire sequencing process and data analysis are carried out within 24 h. Tissue microarray (TMA) has been successfully used in the immunohistochemical study of cervical adenocarcinoma (Tawfik El-Mansi and Williams, 2006). The expression of osteoblast-specific factor 2 in the prostate cancer-related stroma was analyzed by laser capture microdissection of clinical specimens from prostate cancer patients by whole genome expression microarray technology (Wu et al., 2005).

In our study, we selected three metastasis-associated miRNAs from miRNA expression microarray GSE24996. Data mining, network analysis, and experimental validation were applied, and the analytical and experimental results showed potential molecular mechanisms on metastatic melanoma.

## Materials and Methods

### Data Collection

From Gene Expression Omnibus (GEO, https://www.ncbi.nlm.nih.gov/geo/) (Li et al., 2016), we obtained the miRNA expression profile GSE24996 on the basis of the platform of GPL6955 (Agilent-016436 Human miRNA Microarray 1.0). Eight metastatic melanoma samples were screened out and analyzed. We retrieved 15 primary melanoma samples as control.

### Screening for DEMs

For the analysis of DEMs, we first normalized the miRNA expression data through making use of the normalizeBetweenArray function from R package LIMMA (Smyth et al., 2005). Before and after normalizing the data, we showed them in Supplementary Figure 1. Then, the normalized data were used to analyze DEMs through making use of the Limma software package in R software (www.bioconductor.org/packages/release/bioc/html/limma.html) (Kerr, 2003). We set the cut-off value as *P* < 0.05 and |fold change (FC)|>2 for DEM analysis (Li et al., 2016).

### Survival Data From OncoLnc Website

The prognostic values of identified DEMs in metastatic melanoma were obtained on OncoLnc website, which linked TCGA survival data to the expression levels of miRNA, mRNA, or lncRNA(http://www.oncolnc.org/). We thought Log-rank *P* < 0.05 had statistical significance. We selected the survival-associated miRNAs for further study.

### Predicting Target Genes

Through making use of miRTarBase (http://mirtarbase.mbc.nctu.edu.tw/php/index.php), we predicted latent targeted genes of the selected miRNAs. The tool is a microRNA-target interactions database that is experimentally validated (Chou et al., 2018).

### Analysis of GO and Pathway

As a major initiative about bioinformatics, Gene ontology (GO: www.geneontology.org) (Ashburner et al., 2000) encompasses the largest variety of annotations under three headings: cellular component (CC), biological processes (BP), and molecular function (MF). The Kyoto Encyclopedia of Genes and Genomes (KEGG:www.genome.ad.jp/KEGG) (Kanehisa and Goto, 2000) pathway enrichment analysis was applied aiming at investigating the signaling pathways in relation to the unique DEGs. We performed the analysis of GO and KEGG pathway by making use of the Database for Annotation Visualization and Integrated Discovery (DAVID: www.david.ncifcrf.gov/) aiming at identifying the biological significance of DEGs (Huang et al., 2007). We considered *P* < 0.05 as statistical significance.

### Construction of PPI Network and miRNA-Gene Network

We first mapped **three** groups of predicted genes to the Search Tool for the Retrieval of Interacting Genes (STRING) (www.cytoscape.org) (Shannon et al., 2003) to assess functional associations among them, respectively. Next, we analyzed the degree of connectivity in the PPI networks through making use of NetworkAnalyzer module in Cytoscape software and the top 10 higher degree nodes were used as hub genes to construct the miRNA-hub gene network.

### Human Melanoma Samples and Cell Line

We gained 15 metastatic melanoma samples as well as 18 human primary melanoma samples from the Longhua Hospital Affiliated to Shanghai University of Traditional Chinese Medicine. After resection, we froze all specimens in nitrogen, which was liquid at once, and preserved them at −80°C until use. We kept human melanoma cell line SK-MEL-28 (Cat. No. HTB-72, ATCC) in our lab and grew them in 5% CO_2_ in DMEM (Gibco BRL) to which 10% (v/v) FBS (Hyclone) served as a supplement, and we keep the temperature at 37°C.

### Cell Transfection

We transfected the miR-155-5p mimics (HmiR0358-MR04), miR-18a-5p (HmiR-AN0255-AM01) inhibitor, miR-93-5p inhibitor (HmiR-AN0837-AM01), control (CmiR0001-MR04, CmiR-AN0001-AM01) (bought from Fulengen, Guangzhou, China), and si-UBC (bought from Genepharma, Shanghai, China) into SK-MEL-28 cell lines through making use of Lipofectamine™ 2000 on the basis of the instruction of the manufacturer. The sequences were as follows: siUBC-1, 5′-AGAACGUCAAAGCAAAGAU-3′; siUBC-2, 5′-AGAAUGUCAAGGCAAAGAU-3′; siNC, 5′-UUCUCCGAACGUGUCACGUTT-3′.

### Quantitative-PCR (qPCR) and Extraction of RNA

Through introducing RNAiso Plus reagent (Takara Biotechnology Co., Ltd, Dalian), we extracted total RNA from SK-MEL-28 cells and clinical samples. The expression level of miRNA was detected using GeneCopoeia All-in-One™ miRNA qRT-PCR Detection Kit (Cat Nos. AOMD-Q020 or AOMD-Q050) bought from Fulengen Co. Ltd (Guangzhou, China). The primer catalogs were HmiRQP0221 for miR-155-5p, HmiRQP0255 for miR-18a-5p, and HmiRQP0837 for miR-93-5p. Serving as the internal control (HmiRQP9001), the U6 small nuclear RNA was applied. Primers used were as follows: UBC forward (divergent), 5′-GTGTCTAAGTTTCCCCTTTTAAGG-3′ and reverse (divergent), 5′-TTGGGAATGCAACAACTTTATTG-3′; GAPDH forward (divergent), 5′-AGGTCCACCACTGACACGTT-3′; and reverse (divergent), 5′-GCCTCAAGATCATCAGCAAT-3′. Making use of the 2^−ΔΔCT^ method, we aimed at determining fold change in each sample's RNA level in comparison with the sample for reference.

### Transwell Assay

In 24-well transwell chambers (Corning, USA), we conducted the cell invasion experiment. We suspended 1 × 10^5^ transfected cells in 0.2 ml serum-free medium. We added them to the inserts, which were previously coated with Matrigel (BD Bioscience, USA) on the upper surface. Then, we added 0.6 ml medium with 20% FBS to the lower compartment. Forty-eight hours later, after incubation at 37°C, we carefully removed the cells on the membrane's upper surface through making use of a cotton bud. Then we successively fixed cells on the lower surface through making use of 100% methanol and stained them through making use of 0.1% crystal violet. We randomly selected 5 ocular fields of 200 × magnification of each insert. Under a light microscope (Olympus, Japan), we enumerated them.

### Dual Luciferase Assay

The dual luciferase assay used Liofectamine 2,000 to co-transfect UBC 3′-UTR-wt/mut reporter plasmid and miR-93-5p mimic or NC into SK-MEL-28 cells. For 24 h, we used FLUOstar Omega to measure firefly fluorescence Reninase and renin luciferase activity, and renin luciferase activity as a reference. Dual-Lucy Assay Kit (Cat Nos. D0010) was purchased from Beijing Solabo Technology Co., Ltd.

### Western Blot Analysis

We collected 2 groups of SK-MEL-28 cells (miR-93-5p mimic and control). The total protein was extracted by conventional methods, and the protein concentration was determined by the BCA method. After boiled denaturation, we took the same amount of protein-like behavior, SDS-PAGE, pass the membrane, room temperature 5%, milk block, add antibody UBC (Abcam), and incubated overnight. After 30 min of rewarming, TBST was rinsed four times (10 min/time), horseradish peroxidase-labeled two anti-incubated for 1.5 h, and TBST was washed with ECL chemiluminescence method and Quantity One 4.6.2 software for image analysis. β-actin is the reference for this experiment.

### Statistical Analysis

We showed the results as mean ± SD. Through making use of the unpaired Student's *t*-test, we estimated differences between groups. We considered a two-tailed value of *P* < 0.05 as statistical significance.

## Results

### Identification of Survival-Associated DEMs and Their Target Genes

**Figure 1:**
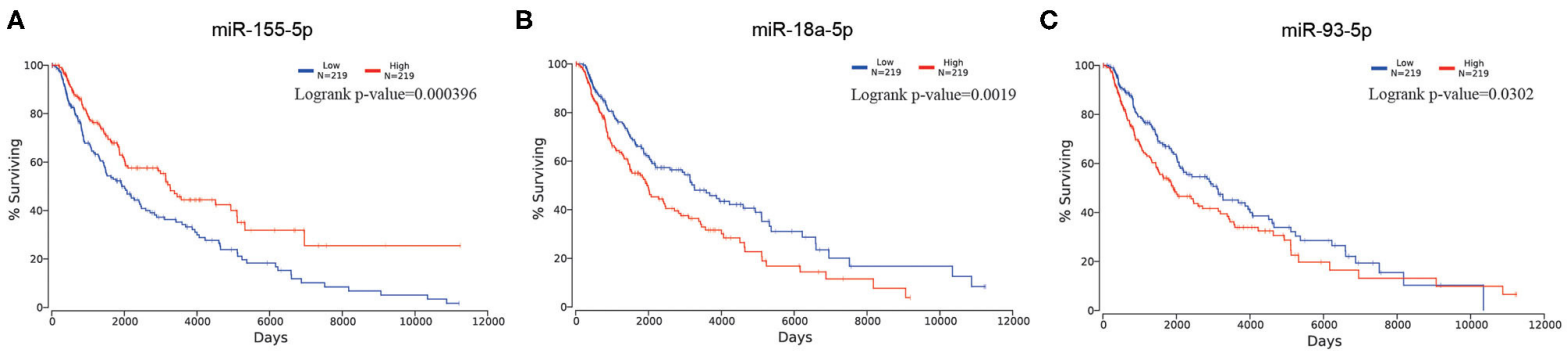
Kaplan–Meier survival curve of selected miRNA in melanoma. **(A)** for miR-155-5p; **(B)** for miR-18a-5p; **(C)** for miR-93-5p.

Aiming at identifying DEMs between samples of metastatic melanoma and samples of primary melanoma, we analyzed the differential expression through making use of limma software package. In total, we identified 63 DEMs that were significantly differentially expressed in metastatic melanoma tissues in comparison with primary melanoma tissues, consisting of 50 upregulated and 13 downregulated miRNAs (Supplementary Table 1). To screen out the survival-associated miRNA, all the 63 DEMs were analyzed on OncoLnc website. Finally, we picked out miR-18a-5p (upregulated), miR-155-5p (downregulated), and miR-93-5p (upregulated) as survival-associated miRNAs (Figure 1).

We predicted 904 miR-155-5p target genes, 262 miR-18a-5p target genes, and 1220 miR-93-5p target genes (Supplementary Table 2).

### GO Function and KEGG Pathway Analysis

**Figure 2:**
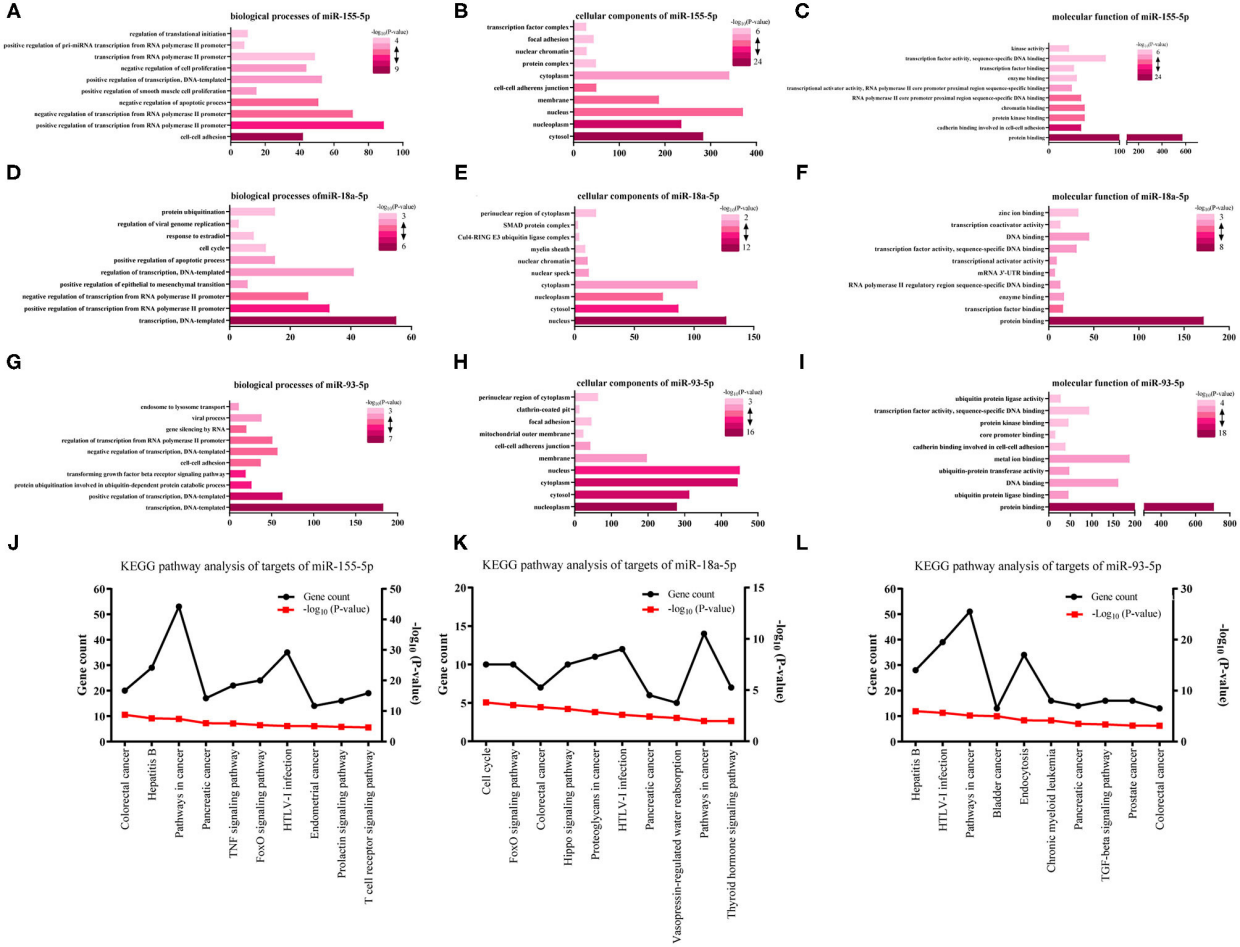
The analysis of GO functions and KEGG pathway for three survival-associated miRNAs target genes. **(A,D,G)** The top 10 enriched biological processes of miR-155-5p, miR-18a-5p, and miR-93-5p; **(B,E,H)** The top 10 enriched cellular components of miR-155-5p, miR-18a-5p, and miR-93-5p; **(C,F,I)** The top 10 enriched molecular function of miR-155-5p, miR-18a-5p, and miR-93-5p. **(J–L)** KEGG pathway analysis for miR-155-5p, miR-18a-5p, and miR-93-5p. The black lines stand for gene count and the red lines stand for–log_10_ (*P*-value).

We analyzed three kinds of GO functional annotation on the three groups of predicted genes, cellular component (CC), molecular function (MF), and biological process (BP) included. The GO BP analysis results demonstrated that miR-155-5p target genes were obviously abundant in cell-cell adhesion, transcription regulated positively/negatively from RNA polymerase II promoter, an apoptotic process regulated negatively, and so on (Figure 2A). The GO CC analysis indicated that miR-155-5p target genes were abundant in membrane, nucleus, nucleoplasm, and cytosol (Figure 2B). The GO MF analysis results indicated that miR-155-5p target genes were obviously abundant in protein binding, cadherin binding participating in protein kinase binding, and cell-cell adhesion (Figure 2C). We showed the GO analysis results of miR-93-5p and miR-18a-5p in Figures 2D–I and the detailed results were presented in Supplementary Table 3.

Aiming at further analyzing the enriched pathways of the three groups of target genes, KEGG pathway enrichment analysis was subsequently conducted (Supplementary Table 4). For miR-155-5p (Figure 2J), the enriched KEGG pathways included colorectal cancer, Hepatitis B, pathways in cancer, and so on. Cell cycle, FoxO signaling pathway, and colorectal cancer, and so on were included in the enriched KEGG pathways for miR-18a-5p (Figure 2K). Hepatitis B, HTLV-I infection, pathways in cancer, and so on were included in the enriched KEGG pathways for miR-93-5p (Figure 2L). Interestingly, FoxO signaling pathway was highlighted in our analysis. As we know, FoxO signaling pathway widely participated in cell autophagy, apoptosis, and proliferation. Whether FoxO signaling pathway participates in melanoma metastasis needs further study.

### Constructing and Analyzing PPI Network and miRNA-Hub Gene Network

We performed the PPI network analysis of three groups of target genes through utilizing the STRING database. We picked out the top 10 hub genes on the basis of the node degree (Table 1). For miR-155-5p, the hub genes were AKT1, EGFR, MYC, CTNNB1, JUN, IL6, PTEN, CCND1, STAT3, and CASP3. For miR-18a-5p, the hub genes were TP53, UBC, CCND1, PTEN, CDC20, ESR1, CCT6A, ATM, SMAD2, and SMAD4. For miR-93-5p, the hub genes were RPS27A, UBC, MYC, GAPDH, HSPA8, MAPK1, PTEN, JUN, CCND1, and POLR2A. The highest node degrees were demonstrated by AKT1, TP53, and UBC among the above genes, which, respectively, were 172, 56, as well as 177. As results showed, AKT1, UBC, and TP53 may serve as pivotal targets in correlation with melanoma metastasis.

**Table 1:** Hub genes identified in the PPI interaction.

**Gene symbol**	**Degree**	**Pathway**
hsa-miR-155-5p		
AKT1	172	Pathways in cancer, TNF signaling pathway, FoxO signaling pathway, Signaling pathways regulating pluripotency of stem cells
EGFR	141	Pathways in cancer, FoxO signaling pathway
MYC	140	Pathways in cancer, Signaling pathways regulating pluripotency of stem cells
CTNNB1	111	Pathways in cancer, Signaling pathways regulating pluripotency of stem cells
JUN	110	Pathways in cancer, TNF signaling pathway, T cell receptor signaling pathway
IL6	107	Pathways in cancer, TNF signaling pathway, FoxO signaling pathway
PTEN	107	Pathways in cancer, FoxO signaling pathway
CCND1	106	Pathways in cancer, FoxO signaling pathway
STAT3	105	Pathways in cancer, FoxO signaling pathway, Signaling pathways regulating pluripotency of stem cells
CASP3	98	Pathways in cancer, TNF signaling pathway
hsa-miR-18a-5p		
TP53	56	Cell cycle, Proteoglycans in cancer, Pathways in cancer, Pathways in cancer, hyroid hormone signaling pathway
UBC	46	NA
CCND1	34	Cell cycle, FoxO signaling pathway, Hippo signaling pathway, Proteoglycans in cancer, Pathways in cancer, Thyroid hormone signaling pathway
PTEN	32	FoxO signaling pathway, Pathways in cancer
CDC20	29	Cell cycle
ESR1	29	Proteoglycans in cancer, Thyroid hormone signaling pathway
CCT6A	21	NA
ATM	21	Cell cycle, FoxO signaling pathway
SMAD2	21	Cell cycle, FoxO signaling pathway, Hippo signaling pathway, Pathways in cancer
SMAD4	20	Cell cycle, FoxO signaling pathway, Hippo signaling pathway, Pathways in cancer
hsa-miR-93-5p		
UBC	177	NA
RPS27A	176	NA
GAPDH	150	NA
MYC	144	Pathways in cancer, TGF-beta signaling pathway
MAPK1	116	Pathways in cancer, TGF-beta signaling pathway
HSPA8	113	NA
PTEN	113	Pathways in cancer
JUN	111	Pathways in cancer
CCND1	104	Pathways in cancer
POLR2A	97	NA

**Figure 3:**
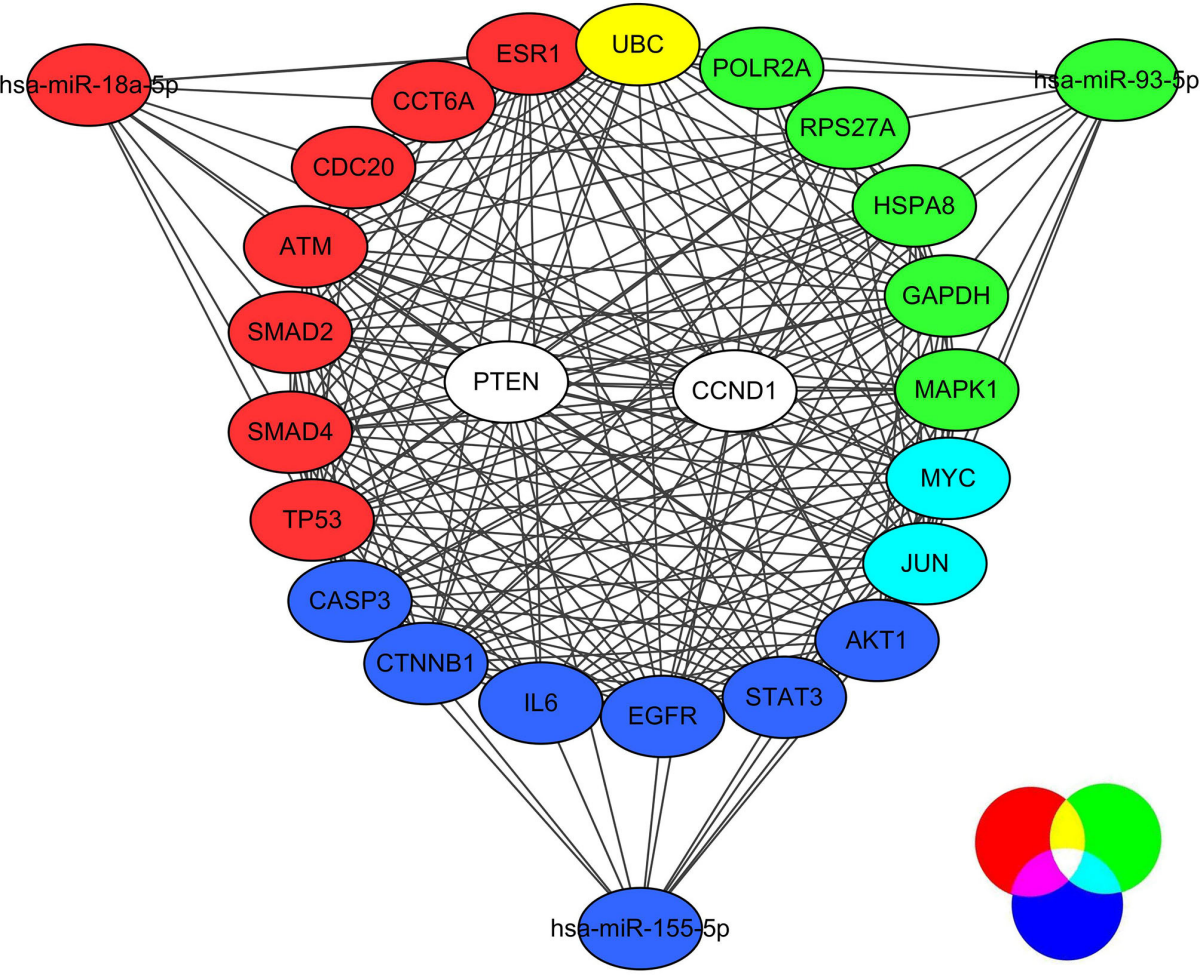
The regulative network between three selected miRNAs and hub genes.

Then, we finished constructing the miRNA-hub gene network by utilizing Cytoscape software. Through miR-18a-5p, MYC and JUN were regulated and miR-93-5p, UBC was regulated through miR-18a-5p and miR-93-5p, PTEN and CCND1 were regulated through the three selected miRNAs, which were all shown in Figure 3. As results showed, PTEN and CCND1 might serve as key targets in correlation with melanoma metastasis.

### Aberrant Expression and *in vitro* Effects of miR-155-5p, miR-18a-5p, and miR-93-5p

**Figure 4:**
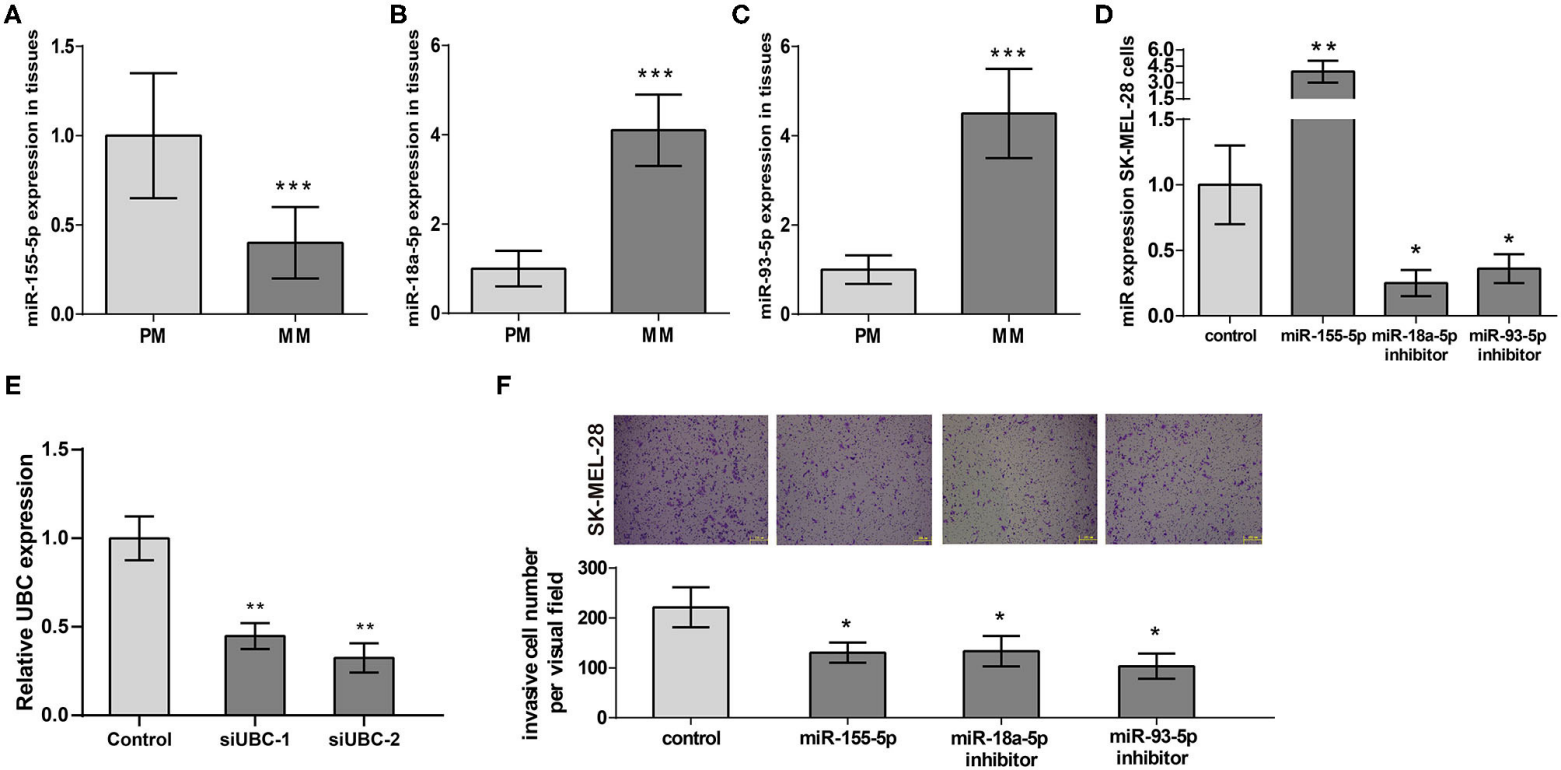
The expression and prognostic functions of miR-155-5p, miR-18a-5p, and miR-93-5p in melanoma metastasis. **(A–C)** miR-155-5p's, miR-18a-5p's, and miR-93-5p's expression in metastatic melanoma tissues (*n* = 15) and primary tissues (*n* = 18). **(D)** Validation of miR-155-5p expression, miR-18a-5p expression, and miR-93-5p expression in transfected SK-MEL-28 cells. **(E)** Validation of UBC expression in transfected SK-MEL-28 cells. **(F)** SK-MEL-28 transfected with miR-155-5p, miR-18a-5p inhibitor, or miR-93-5p inhibitor invaded less vs. control cancer cells. ***P < 0.001, **P < 0.01, *P < 0.05. PM, primary melanoma; MM, metastatic melanoma.

Following our miRNA expression profile analysis, miR-155-5p's, miR-93-5p's, and miR-18a-5p's expression at 18 PM and 15 MM samples were verified. We presented the results in Figures 4A–C that demonstrated that miR-155-5p expression was obviously downregulated, miR-18a-5p and miR-93-5p expression were obviously regulated positively in MM samples in comparison with PM samples, which verified the analysis results in miRNA expression microarray.

Next, we transfected miR-155-5p expression plasmid, miR-18a-5p inhibitor plasmid, and miR-93-5p inhibitor plasmid into melanoma cell line SK-MEL-28, respectively. RT-qPCR results exhibited miR-155-5p's overexpression in SK-MEL-28 and miR-93-5p and miR-18a-5p's downregulation in SK-MEL-28 (Figure 4D). In addition, RT-qPCR results showed that in SK-MEL-28 transfected with si-UBC, the expression level of UBC was reduced (Figure 4E). Then, we employed a transwell invasion assay aiming at measuring the effects of miR-155-5p, miR-18a-5p, and miR-93-5p on melanoma invasion. The results shown in Figure 4F indicated that miR-155-5p's upregulation of miR-93-5p's and miR-18a-5p's downregulation could, respectively, suppress SK-MEL-28 cell invasion. All of the above data proved that miR-155-5p, miR-18a-5p, and miR-93-5p were key regulators of melanoma metastasis. Targeting miR-155-5p, miR-18a-5p, and miR-93-5p to regulate the invasion of melanoma cells may represent novel approaches in treating melanoma patients.

### Inhibition of miR-93-5p Could Reduce SK-MEL-28 Cells Invasive Ability Through UBC

**Figure 5:**
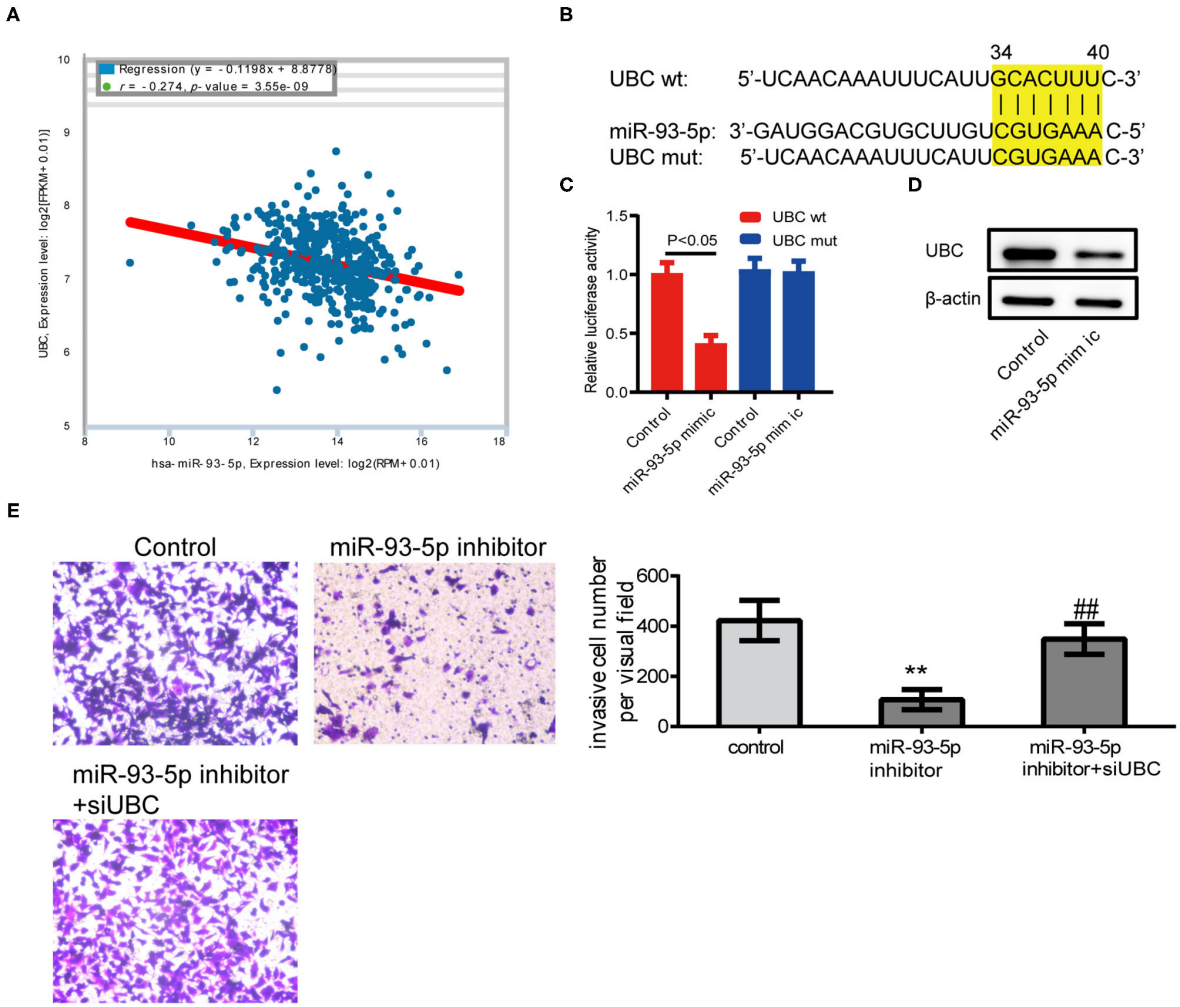
Inhibition of miR-93-5p could reduce SK-MEL-28 cells' invasive ability through UBC. **(A)** The miR-93-5p and UBC expression data of 449 melanoma patients from ENCORI. We observed the inverse correlation with statistical significance between miR-93-5p and UBC mRNA. **(B)** Diagram of UBC 3'UTR including 1 putative conserved target sites for miR-93-5p, recognized through making use of the TargetScan database. **(C)** Results of luciferase reporter experiments in SK-MEL-28 cells, with co-transfection of wt or mt 3'UTR and miR mimic, as shown. **(D)** The protein expression of UBC in SK-MEL-28 cells transfected with miR-93-5p. **(E)** SK-MEL-28 transfected with miR-93-5p inhibitor invaded less vs. control cancer cells, and the anti-invasive effect by miR-93-5p inhibitor was reduced when the expression of UBC was decreased by its siRNA. **P < 0.01 miR-93-5p inhibitor vs. control, ^##^*P* < 0.01 miR-93-5p inhibitor vs. miR-93-5p inhibitor+siUBC.

According to the results of previous studies, there is no related report of miR-93-5p in melanoma. To validate the results of comprehensive analysis, we picked out miR-93-5p and its potential target gene UBC for further study. Using the expression data of melanoma from ENCORI (http://starbase.sysu.edu.cn/), it was found that miR-93-5p expression was obviously in inverse correlation with UBC in 449 melanoma patients (Figure 5A). The predicted site in UBC 3′-UTR that can be bound by miR-93-5p is illustrated in Figure 5B. We consequently explored whether UBC served as miR-93-5p's direct target in SK-MEL-28 cells. The luciferase reporter experiment indicated that miR-93-5p mimic obviously inhibited the luciferase activity in SK-MEL-28 cells with wt-UBC-3′UTR vector, but not in mutant UBC-3′ UTR (Figure 5C). Moreover, transfected miR-93-5p mimic gave rise to an obvious reduction of UBC protein (Figure 5D). To verify whether the reduction of SK-MEL-28 cells' invasive ability by inhibiting miR-93-5p was dependent on UBC, siRNA targeting UBC was transfected into miR-93-5p-upregulating SK-MEL-28 cells. The results showed that the anti-invasive effect by miR-93-5p's downregulation was reduced when the expression of UBC was decreased by its siRNA (Figure 5E).

## Discussion

Poor outcome of melanoma mainly results from metastasis. Until now, the mechanism of melanoma metastasis remained unclear. Through previous studies and reports, it was found that most primary melanomas can be cured by local resection (Damato, 2012), but metastatic melanomas have historically had a poor prognosis, with a median survival time of 9 months and a long-term survival rate of 10% (Hall et al., 2000). Metastatic melanoma accounts for approximately 80% of skin cancer-related deaths (Aladowicz et al., 2013). The more understanding of the pathogenesis of metastasis, the better we can develop drugs and treat this disease. Increasing evidence has shown that miRNA expression profiling analysis is a useful tool to study cancer progression and metastasis. Abundant miRNAs took part in numerous pivotal cellular pathways in relation to the progression of cancer, which we have recognized to be expressed aberrantly in melanoma.

**Figure 6:**
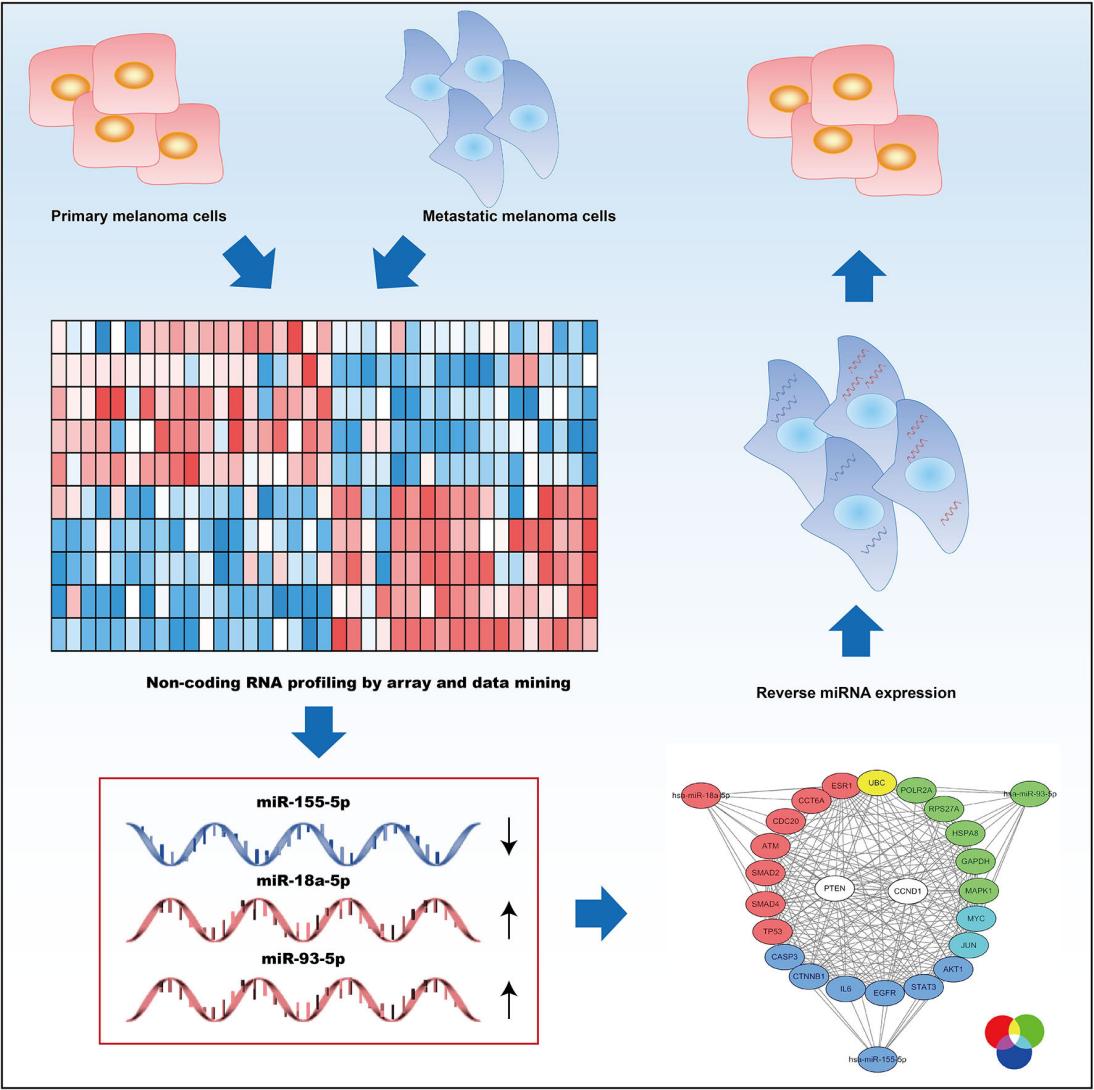
A schematic illustration of the metastasis-associated microRNAs in melanoma.

Up to now, we have done the research on miRNA associated with metastasis for melanoma by miRNA expression profiling, whose values are relatively limited (Figure 6). As for the research, we gathered melanoma's miRNA expression profiling dataset and systemically analyzed them, aiming at retrieving miRNAs associated with metastasis. A total of 63 DEMs were identified, many of which were known to be involved in melanoma. For example, melanoma cell-secreted exosomal miR-155-5p, which was selected as a survival-associated miRNA, could induce a proangiogenic switch of fibroblasts associated with cancer through improving proangiogenic factors' expression in recipient fibroblasts via SOCS1/JAK2/STAT3 signaling pathway (Zhou et al., 2018). MiR-141-3p, which was regulated negatively in our study, showed the inhibitory effects on melanoma cells' anchorage-independent growth ability, their potential of invasion, and expression of an embryonic-like, multipotent, aggressive cancer phenotype. Further data suggested that vasculogenic mimicry was regulated by miR-141-3p through phosphatidyl inositol-3-kinase (PI3K) (Verrando et al., 2016) and extracellular signal-regulated kinase 1/2 (ERK1/2). MiR-330-3p inhibited melanoma proliferation by targeting TRX2 (Yao et al., 2018). miR-198, regulated by hsa_circ_0025039, inhibited cell growth, glucose metabolism, and invasion in malignant melanoma via targeting CDK4 (Bian et al., 2018).

Furthermore, the building of the PPI network was finished on the basis of the predicted genes. Additionally, we made use of analyses of GO and KEGG pathway enrichment aiming at further interpreting their functions about biology. Then, we identified the top 10 hub nodes whose degrees were higher for each miRNA as hub genes. AKT1, TP53, and UBC had the highest node degrees among these genes. Through miRNA-hub gene network construction, it was found that PTEN and CCND1 were regulated by all three miRNAs. The results indicated that AKT1, TP53, UBC, PTEN, and CCND1 may be pivotal targets in correlation with melanoma metastasis. Previous studies suggested that AKT1 promoted the development of melanoma metastases (Cho et al., 2015), and its melanoma-derived mutation, AKT1^E17K^, activated focal adhesion kinase and promoted melanoma brain metastasis (Kircher et al., 2019). In addition, p53, encoding by TP53 gene, was reported as a therapeutic target of melanoma (Wu et al., 2018). The other three key genes, UBC, PTEN, and CCND1, were also well studied in melanoma (Roh et al., 2016; Donigan et al., 2017; Uguen et al., 2017; Mu and Sun, 2018; Zhang et al., 2018; Zhu et al., 2018; Chen et al., 2019; Giles et al., 2019). Overall, systematically analyzing miRNA profiling took a step in the investigation of the mechanisms that underlay melanoma's metastasis.

In future studies, we will collect more clinical samples to explore the prognostic value of miR-18a-5p, miR-155-5p, and miR-93-5p in melanoma. In addition, we will further study the mechanism of miR-93-5p/UBC *in vivo*.

To sum up, through analyzing the miRNA expression profile from an open database, many biological miRNAs were identified, which may participate in melanoma's metastasis. This work verified the function of miR-155-5p, miR-18a-5p, and miR-93-5p in melanoma metastasis, providing additional insights into the complex process of this disease. Meanwhile, we identified miR-93-5p as a pro-invasion miRNA by regulating the expression of UBC in melanoma for the first time. Our research found that miR-18a-5p, miR-155-5p, and miR-93-5p play a key role in the mechanism of melanoma metastasis, and proved that miR-93-5p/UBC is a potential effective target for melanoma.

## Data Availability Statement

The original contributions presented in the study are included in the article/Supplementary Material, further inquiries can be directed to the corresponding author/s.

## Ethics Statement

The studies involving human participants were reviewed and approved by The ethics committee of Longhua Hospital Affiliated to Shanghai University of Traditional Chinese. The patients/participants provided their written informed consent to participate in this study.

## Author Contributions

YG, JX, and HL: conception and design. YG, YH, and GY: development of methodology. GY and YG: analysis and interpretation of data. YG, JX, HL, YH, and GY: writing, review, and/or revision of the manuscript.

## Conflict of Interest

The authors declare that the research was conducted in the absence of any commercial or financial relationships that could be construed as a potential conflict of interest.

## Supplementary Material

Supplementary Figure 1 Before and after the normalization of the miRNA expression data using the normalize BetweenArray function from R package LIMMA.

Supplementary Table 1 List of differentially expressed miRNAs between MM samples and PM samples.

## References

[B1] Aladowicz E. Ferro L. Vitali G. C. Venditti E. Fornasari L. Lanfrancone L. (2013). Molecular networks in melanoma invasion and metastasis. Future Oncol. 9, 713–726. 10.2217/fon.13.9 23647299

[B2] Ashburner M. Ball C. A. Blake J. A. Botstein D. Butler H. Cherry J. M.. (2000). Gene ontology: tool for the unification of biology. The Gene Ontology Consortium. Nat. Genetics 25, 25–29. 10.1038/75556 10802651 PMC3037419

[B3] Azoury S. C. Lange J. R. (2014). Epidemiology, risk factors, prevention, and early detection of melanoma. Surg. Clin. North Am. 94, 945–962. 10.1016/j.suc.2014.07.013 25245960

[B4] Bakkal F. K. Basman A. Kizil Y. Ekinci O. Gumusok M. Ekrem Zorlu M.. (2015). Mucosal melanoma of the head and neck: recurrence characteristics and survival outcomes. Oral Surg. Oral Med. Oral Pathol. Oral Radiol. 120, 575–580. 10.1016/j.oooo.2015.06.038 26260765

[B5] Barrett T. Wilhite S. E. Ledoux P. Evangelista C. Kim I. F. Tomashevsky M.. (2013). NCBI GEO: archive for functional genomics data sets–update. Nucleic Acids Res. 41, D991–D995. 10.1093/nar/gks1193 23193258 PMC3531084

[B6] Bian D. H. Wu Y. Song G. D. (2018). Novel circular RNA, hsa_circ_0025039 promotes cell growth, invasion and glucose metabolism in malignant melanoma via the miR-198/CDK4 axis. Biomed. Pharmacother. 108, 165–176. 10.1016/j.biopha.2018.08.152 30219673

[B7] Chen J. Wu F. Shi Y. Yang D. Xu M. Lai Y.. (2019). Identification of key candidate genes involved in melanoma metastasis. Mol. Med. Rep. 20, 903–914. 10.3892/mmr.2019.10314 31173190 PMC6625188

[B8] Chien W. W. Sun Q. Y. Lee K. L. Ding L. W. Wuensche P. Torres-Fernandez L. A.. (2015). Activation of protein phosphatase 2A tumor suppressor as potential treatment of pancreatic cancer. Mol. Oncol. 9, 889–905. 10.1016/j.molonc.2015.01.002 25637283 PMC4387089

[B9] Cho J. H. Robinson J. P. Arave R. A. Burnett W. J. Kircher D. A. Chen G.. (2015). AKT1 activation promotes development of melanoma metastases. Cell Rep. 13, 898–905. 10.1016/j.celrep.2015.09.057 26565903 PMC4646731

[B10] Chou C. H. Shrestha S. Yang C. D. Chang N. W. Lin Y. L. Liao K. W.. (2018). miRTarBase update 2018: a resource for experimentally validated microRNA-target interactions. Nucleic Acids Res. 46, D296–D302. 10.1093/nar/gkx1067 29126174 PMC5753222

[B11] Damato B. E. (2012). Local resection of uveal melanoma. Dev. Ophthalmol. 49, 66–80. 10.1159/000328261 22042014

[B12] Donigan J. M. De Luca J. Lum C. (2017). Cyclin D1 and p16 expression in blue nevi and malignant melanoma. Appl Immunohisto M M. 25, 91–94. 10.1097/PAI.0000000000000276 26766120

[B13] Giles K. M. Rosenbaum B. E. Berger M. Izsak A. Li Y. Bochaca I. I.. (2019). Revisiting the clinical and biologic relevance of partial PTEN loss in melanoma. J. Invest. Dermatol. 139, 430–438. 10.1016/j.jid.2018.07.031 30148988 PMC6342667

[B14] Hall W. A. Djalilian H. R. Nussbaum E. S. Cho K. H. (2000). Long-term survival with metastatic cancer to the brain. Med. Oncol. 17, 279–286. 10.1007/BF02782192 11114706

[B15] Harris T. J. McCormick F. (2010). The molecular pathology of cancer. Nat. Rev. Clin. Oncol. 7, 251–265. 10.1038/nrclinonc.2010.41 20351699

[B16] Hu N. Clifford R. J. Yang H. H. Wang C. Y. Goldstein A. M. Ding T.. (2010). Genome wide analysis of DNA copy number neutral loss of heterozygosity (CNNLOH) and its relation to gene expression in esophageal squamous cell carcinoma. BMC Genomics 11:576. 10.1186/1471-2164-11-576 20955586 PMC3091724

[B17] Huang D. W. Sherman B. T. Tan Q. Collins J. R. Alvord W. G. Roayaei J.. (2007). The DAVID gene functional classification tool: a novel biological module-centric algorithm to functionally analyze large gene lists. Genome Biol. 8:R183. 10.1186/gb-2007-8-9-r183 17784955 PMC2375021

[B18] Kanavy H. E. Gerstenblith M. R. (2011). Ultraviolet radiation and melanoma. Seminars Cutaneous Med. Surg. 30, 222–228. 10.1016/j.sder.2011.08.003 22123420

[B19] Kanehisa M. Goto S. (2000). KEGG: kyoto encyclopedia of genes and genomes. Nucleic acids Res. 28, 27–30. 10.1093/nar/28.1.27 10592173 PMC102409

[B20] Kerr M. K. (2003). Linear models for microarray data analysis: hidden similarities and differences. J. Comp. Biol. 10, 891–901. 10.1089/106652703322756131 14980016

[B21] Kircher D. A. Trombetti K. A. Silvis M. R. Parkman G. L. Fischer G. M. Angel S. N.. (2019). AKT1(E17K) activates focal adhesion kinase and promotes melanoma brain metastasis. Mol. Cancer Res. 17, 1777–1786. 10.1158/1541-7786.MCR-18-1372 31138602 PMC6726552

[B22] Li J. Y. Zheng L. L. Wang T. T. Hu M. (2016). Functional annotation of metastasis-associated MicroRNAs of melanoma: a meta-analysis of expression profiles. Chinese Med. J. 129, 2484–2490. 10.4103/0366-6999.191793 27748342 PMC5072262

[B23] Lou W. Chen J. Ding B. Chen D. Zheng H. Jiang D.. (2018). Identification of invasion-metastasis-associated microRNAs in hepatocellular carcinoma based on bioinformatic analysis and experimental validation. J. Translational Med. 16:266. 10.1186/s12967-018-1639-8 30268144 PMC6162949

[B24] McComiskey M. Iavazzo C. Datta M. Slade R. Winter-Roach B. Lambe G.. (2015). Balloon cell urethral melanoma: differential diagnosis and management. Case Rep. Obstet. Gynecol. 2015:919584. 10.1155/2015/919584 26257971 PMC4516829

[B25] Mu Z. Sun Q. (2018). Cantharidin inhibits melanoma cell proliferation via the miR-21-mediated PTEN pathway. Mol. Med. Rep. 18, 4603–4610. 10.3892/mmr.2018.9440 30221692

[B26] Roh M. R. Gupta S. Park K. H. Chung K. Y. Lauss M. Flaherty K. T.. (2016). Promoter methylation of PTEN is a significant prognostic factor in melanoma survival. J. Invest. Dermatol. 136, 1002–1011. 10.1016/j.jid.2016.01.024 26854490

[B27] Shaikh W. R. Dusza S. W. Weinstock M. A. Oliveria S. A. Geller A. C. Halpern A. C. (2016). Melanoma thickness and survival trends in the United States, 1989 to 2009. J. Natl. Cancer Inst. 108:djv294. 10.1093/jnci/djv294 26563354 PMC4857148

[B28] Shannon P. Markiel A. Ozier O. Baliga N. S. Wang J. T. Ramage D.. (2003). Cytoscape: a software environment for integrated models of biomolecular interaction networks. Genome Res. 13, 2498–2504. 10.1101/gr.1239303 14597658 PMC403769

[B29] Smyth G. K. Michaud J. Scott H. S. (2005). Use of within-array replicate spots for assessing differential expression in microarray experiments. Bioinformatics 21, 2067–2075. 10.1093/bioinformatics/bti270 15657102

[B30] Tabaries S. Ouellet V. Hsu B. E. Annis M. G. Rose A. A. N. Meunier L.. (2015). Granulocytic immune infiltrates are essential for the efficient formation of breast cancer liver metastases. Breast Cancer Res. 17:45. 10.1186/s13058-015-0558-3 25882816 PMC4413545

[B31] Tawfik El-Mansi M. Williams A. R. (2006). Validation of tissue microarray technology using cervical adenocarcinoma and its precursors as a model system. Int. J. Gynecol. Cancer 16, 1225–1233. 10.1111/j.1525-1438.2006.00570.x 16803510

[B32] Uguen A. Guibourg B. Uguen M. (2017). Another point of view about cyclin D1 and p16 expression in blue nevi and malignant melanomas. Appl. Immunohisto M M 25, E70–E1. 10.1097/PAI.0000000000000431 27490765

[B33] Verrando P. Capovilla M. Rahmani R. (2016). Trans-nonachlor decreases miR-141-3p levels in human melanocytes *in vitro* promoting melanoma cell characteristics and shows a multigenerational impact on miR-8 levels in Drosophila. Toxicology 368, 129–141. 10.1016/j.tox.2016.09.003 27616325

[B34] Wu C. E. Esfandiari A. Ho Y. H. Wang N. Mahdi A. K. Aptullahoglu E.. (2018). Targeting negative regulation of p53 by MDM2 and WIP1 as a therapeutic strategy in cutaneous melanoma. Brit. J. Cancer 118, 495–508. 10.1038/bjc.2017.433 29235570 PMC5830592

[B35] Wu M. S. Lin Y. S. Chang Y. T. Shun C. T. Lin M. T. Lin J. T. (2005). Gene expression profiling of gastric cancer by microarray combined with laser capture microdissection. World J. Gastroenterol. 11, 7405–7412. 10.3748/wjg.11.7405 16437709 PMC4725172

[B36] Yao Y. Zuo J. Wei Y. G. (2018). Targeting of TRX2 by miR-330-3p in melanoma inhibits proliferation. Biomed. Pharmacother. 107, 1020–1029. 10.1016/j.biopha.2018.08.058 30257313

[B37] Zhang J. Liu W. L. Zhang L. Ge R. He F. Gao T. Y.. (2018). MiR-637 suppresses melanoma progression through directly targeting P-REX2a and inhibiting PTEN/AKT signaling pathway. Cell Mol Biol. 64, 50–57. 10.14715/cmb/2018.64.11.10 30213289

[B38] Zhou X. Yan T. Huang C. Xu Z. Wang L. Jiang E.. (2018). Melanoma cell-secreted exosomal miR-155-5p induce proangiogenic switch of cancer-associated fibroblasts via SOCS1/JAK2/STAT3 signaling pathway. J. Exp. Clin. Cancer Res. 37:242. 10.1186/s13046-018-0911-3 30285793 PMC6169013

[B39] Zhu Y. Wen X. Zhao P. (2018). MicroRNA-365 inhibits cell growth and promotes apoptosis in melanoma by targeting BCL2 and cyclin D1 (CCND1). Med. Sci. Monitor. 24, 3679–3692. 10.12659/MSM.909633 29858490 PMC6011806

