# Supplementary figures and images for: Identification of Metastasis-Associated MicroRNAs in Metastatic Melanoma by miRNA Expression Profile and Experimental Validation
Source: Front Genet. 2021 Apr 9;12:663110. doi: 10.3389/fgene.2021.663110 (PMC8063111; doi:10.3389/fgene.2021.663110)

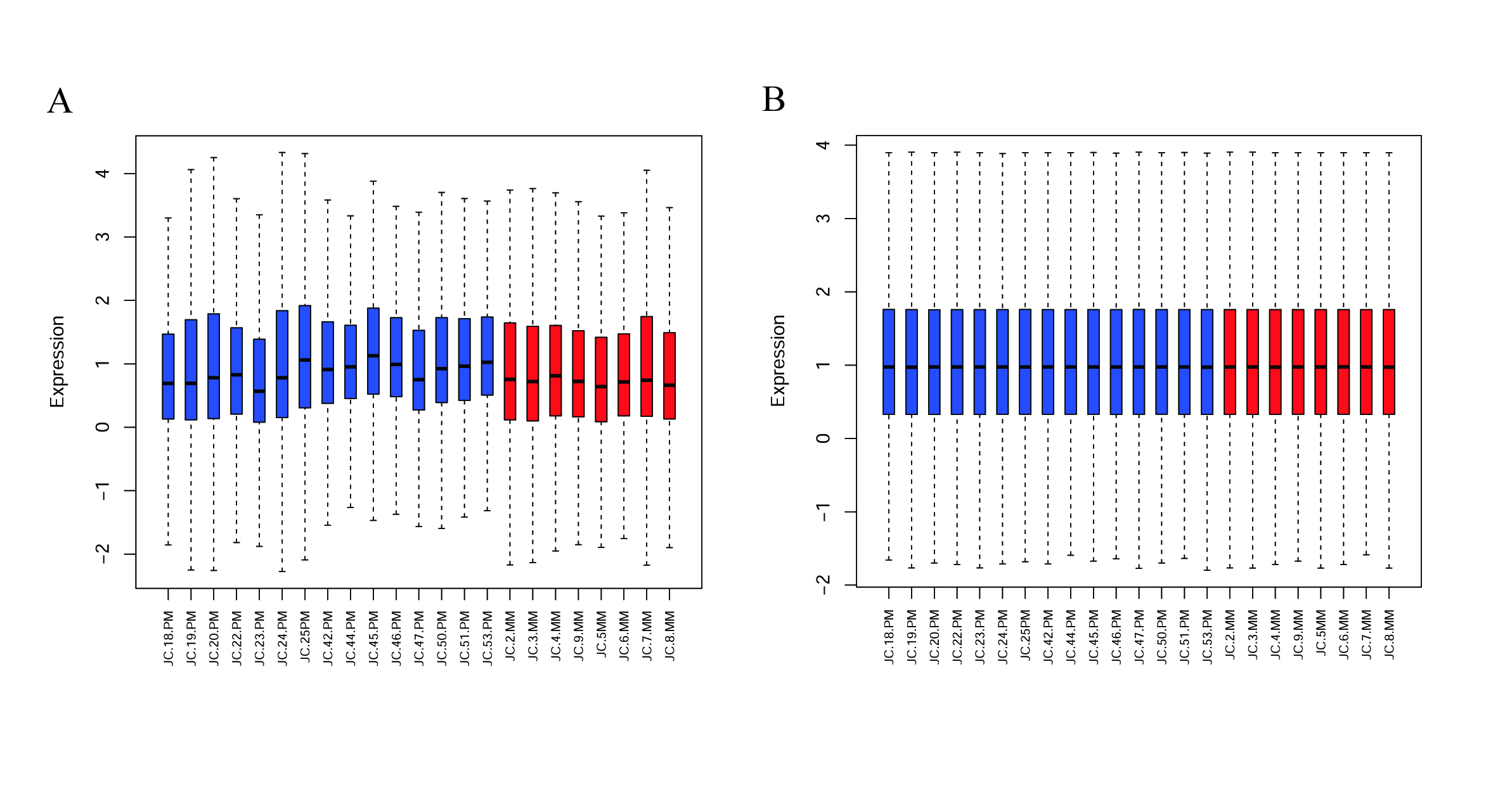

Supplement: Supplementary Figure 1 — Before and after the normalization of the miRNA expression data using the normalize BetweenArray function from R package LIMMA. [file Image_1.JPEG]
